# PROPRIOCEPTION, BODY BALANCE AND FUNCTIONALITY IN INDIVIDUALS WITH ACL RECONSTRUCTION

**DOI:** 10.1590/1413-785220162402108949

**Published:** 2016

**Authors:** Tássia Silveira Furlanetto, Leonardo Alexandre Peyré-Tartaruga, Alexandre Severo do Pinho, Emanuele da Silva Bernardes, Milton Antonio Zaro

**Affiliations:** 1. Universidade Federal do Rio Grande do Sul (UFRGS), Porto Alegre, RS, Brazil; 2. Universidade Federal de Ciências da Saúde de Porto Alegre (UFCSPA), Porto Alegre, RS, Brazil; 3. Instituto Brasileiro de Couro, Calçados e Artefatos (IBTeC), Novo Hamburgo, RS, Brazil

**Keywords:** Anterior cruciate ligament, Proprioception, Postural balance, Knee

## Abstract

**Objective:**

: To evaluate and compare proprioception, body balance and knee functionality of individuals with or without unilateral anterior cruciate ligament (ACL) reconstruction.

**Methods:**

: Forty individuals were divided in two groups: Experimental group, 20 individuals with ACL reconstruction at six months postoperative, and control group, 20 individuals with no history of lower limb pathologies. In the experimental group, we assessed lower limbs with reconstructed ACL and contralateral limb; in the control group the dominant and the non-dominant lower limbs were assessed. All subjects were submitted to joint position sense test to evaluate proprioception, postural control measure in single-limb, and step up and down (SUD) test for functional assessment.

**Results:**

: There were no deficits in proprioception and postural control. In the SUD test, a 5% decrease in lift up force was found in reconstructed ACL lower limbs, however, a statistically not significant difference. The impact and step down force during the course of test were 30% greater in anatomic ACL than in control lower limbs.

**Conclusion:**

: The individuals with ACL reconstruction at six months postoperative did not show changes in proprioception and postural control, but showed motor control changes, influencing knee functionality. Level of Evidence IV, Prognostic Studies.

## INTRODUCTION

The anterior cruciate ligament (ACL) is one of the major ligaments providing mechanical stability of the knee, controlling the anteroposterior translation and rotation movements, playing a key role in neuromuscular stability, since it is involved in the articular movement sensory feedback, thereby contributing to proprioception.^1^^-^ 3 Proprioception includes afferent and efferent path of the somatosensory system controlling reflexes and muscle tone of muscles, tendons and articulations.^1^ The efferent innervation is given by nerve fibers penetrating the cruciate ligaments and it is based in afferent mechanoreceptors located in peripheral joints, muscles and skin.^4^ At the ACL, they represent between 1 and 2% of the volume.^3^

The ACL is affected in more than 50% of ligament injuries, and the complete breakdown of the fibers cause removal of mechanoreceptors present in the joint.^5^^,^ 6 The decrease of sensory information after ACL injury alters the afferent information to the central nervous system (CNS), influencing sensitivity, impairing the ability to detect motion and inhibiting muscle motor neurons that surround the joint,^7^^,^ 8 changing the motor control of the lower limbs (LLs).^9^^,^ 10

The total ACL rupture causes limitations of joint movements, mechanical and functional instability of the anterolateral knee, loss of force, muscle imbalance, atrophy and impaired neuromuscular function.^8^^,^ 11 ^,^ 12 Because of these changes, the ACL reconstruction surgery is often recommended and, together with appropriate rehabilitation, it is expected to improve the static stability and restore knee functionality by reinforcement of neuromuscular control.^8^

There is no consensus about the time of post-surgical recovery, varying from five to twelve months after the reconstruction.^4^ The proposed recommendation in rehabilitation clinics involves follow-up for a period up to six months after surgery,^13^^,^ 14 although there is a possibility that patients show stability and functionality deficits up to two years post-surgery.^8^

Integrity of the ACL is critical to knee functionality and it is often analyzed through functional activity tests or muscle force evaluation.^14^ However, the literature reports the use of functional high impact tests, which are not advised for non-athlete individuals or after a short post surgical period.^15^ Moreover, evaluation of muscle force does not represent a functional activity of daily living. An alternative would be to measure force alterations during activities that represent those of daily living.^16^

Sensory and motor deficits can be found in individuals with ACL reconstruction, but there are many differences in study results. Moreover, there is still a gap in the literature on post-surgical rehabilitation time and the existence of clinical evaluations of ACL injuries for non-athlete individuals recommended for monitoring the patients' different postoperative periods. The objective of this study was to evaluate and compare proprioception, postural control and knee function in subjects with and without unilateral ACL reconstruction. 

## MATERIALS AND METHODS

The sample size was determined by conducting a sample calculation using the Epidemiologic Perspectives & Innovations Program (EPIP) version 1.4 (US), adopting a 0.05 significance level and correlation power of 80%, based on previous studies.^8^^,^ 17 Thus, the sample consisted of 40 subjects, divided into two groups: experimental group (EG) and control group (CG). The EG consisted of 20 subjects, with a mean age of 29.2 ± 8.1 years old, mean body mass 81.2 ± 14.4 kg and mean height 173.1 ± 7.5 cm. Inclusion criteria for the EG were having unilateral ACL reconstruction surgery with a postoperative period of six months and not having any other injury in the LLs. The CG consisted of 20 subjects with a mean age of 27.8 ± 4.0 years old, mean body mass 70.3 ± 14.2 kg and mean height 170.8 ± 8.8 cm. In the control group only individuals without history of LLs injury in the past five years were included.

In the EG the LLs with reconstructed ACL (ACLr) and its healthy contralateral limb (ACLa) were evaluated. In the CG, the LLs were named according to the dominance: not dominant (NDLL) and dominant (DLL). All subjects signed a Free and Informed Consent Term, authorizing participation in the study. This study was approved by the Ethics Committee of the institution involved in the study.

For data acquisition, each individual went through the following procedures: 1) joint position sense test (JPS) of the knee; 2) evaluation of unipodal postural control (PC); and 3) step up and down (SUD) test. All unilateral procedures were performed for both LLs.

**Figure 1.**
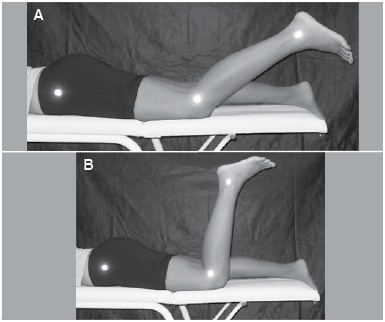
Joint position sense test for (A) 40° knee flexion; (B) 90° knee flexion.

To carry out the JPS test, which evaluates proprioception, anatomical reference points were identified: greater trochanter of the femur, articular line of the knee and lateral malleolus. The individual was placed in the prone position, without any visual contact with the LLs. The evaluator conducted the knee flexion movement passively, with the aid of a goniometer, and immediately after, the individual should have actively repeated the position. The test was performed at 90° and 40° knee flexion. (Figure 1) Positions were recorded with digital images using a 4.1 megapixels digital camera (model L100, Samsung, Japan) coupled to a 96 cm tripod, 1.65 m horizontally apart from the individual. The protocol was repeated three times for each position.

For the evaluation of PC and to perform the SUD test we used two-dimensional force platforms (AMTI/OR6-7) arranged in parallel, named P1 and P2. Data were normalized by the individuals' body weight.

**Figure 2.**
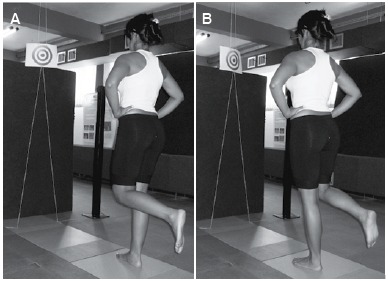
Assessment of postural control for (A) right unipodal support; (B) left unipodal support.

The collection of PC data was performed in the semi-static position at a sampling rate of 100 Hz. Three records of each position (right and left unipodal support), lasting for 30 sec each. The individual was asked to remain still in the indicated position with his/her hands on the anterosuperior iliac crests (ASIC), silently, gaze fixed on a target, located 1m from the eye level of each participant. (Figure 2)

**Figure 3.**
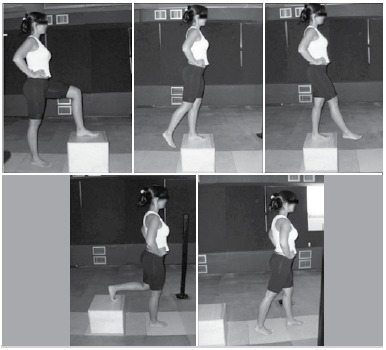
Step up and down test to assess functionality.

The collecting of data from SUD test, used to evaluate functionality, occurred at a sampling rate of 2000 Hz. The step corresponded to a 30 cm high wooden box placed on P1. The test started off the force platform with the patient in static position, legs together and hands on ASIC. The individual climbed the step (P1) with one of his/her LLs and descended it by stepping on P2 in a continuous single movement. The test was performed five times with each starting LL. (Figure 3)

The JPS test was analyzed by photogrammetry, using the Postural Assessment Software (PAS), as previously validated.^18^ The 40° and 90° knee bending angle (KBA) was examined using scanning points. These angles were measured on the passive movement image (performed by the evaluator) and the active movement (performed by the individual). Later on, the KBA difference between the passive and the active position (DIF) of each LL was calculated, allowing to determine whether the individual had a good sense of knee joint position.

To measure PC, the amplitudes of center of pressure (COP) in the anteroposterior (COPap) and medial-lateral (COPml) directions were analyzed. The maximum amplitude of COPap and COPml were recorded in each condition.

To assess the SUD test, the curves of the vertical component of the ground reaction force (Fz) exerted on climbing and descending the step were calculated and analyzed. The first Fz peak of each curve was calculated, normalized by the body weight of the individual and presented in percentage. The force data were filtered by fourth order digital low-pass Butterworth filter with cutoff frequency 12 Hz. Moreover, the impact exerted on each LL descending the step was calculated through the load application rate (LAR). LAR is associated with the slope of the curve (force x time) during the step descending period. High LAR levels indicated that the locomotor apparatus was under these forces for a short time, characterizing a large impact, while smaller values indicate that forces were distributed over a greater time interval, thereby reducing impact. The maximum LAR of the first peak of the Fz curve descending the step was calculated.

Statistical analysis was performed using SPSS 19.0 software (US). Initially the normality of the data was verified (Shapiro-Wilk) and variance homogeneity (Levene test) 

According to descriptive analysis, parametric data are expressed as mean ± standard deviation and non-parametric data as median ± standard error. For inferential analysis, it was initially verified whether there was a difference between the LLs of the CG: DLL and NDLL. For parametric data we used the paired Student's *t-* test and for non-parametric data, the Wilcoxon test. As there was no significant difference for any of the variables, the LL of the CG was chosen at random and named control lower limb (CLL). 

For comparison among the three groups (ACLr, ACLa and CLL) we used one-way ANOVA for parametric data, with post-hoc Tukey test. In case of non-parametric distribution, the Kruskal-Wallis test was used, and to find the differences, the Mann-Whitney test was used. The significance level was set at α = 0.05.

## RESULTS

Table 1 shows the median values ± standard error of KBA found in passive and active movements, for both the positions 40° and 90°. There was no significant difference between the DIF values between ACLr ACLa and CLL groups, for positions 40° (p = 0.883) and 90° (p = 0.385). These findings show that, regardless the injury, the individual can perceive the LL position, passively determined by the evaluator, and actively repeat the movement.

**Table 1.** Median ± standard error values of KBA in positions 40° and 90° for active and passive movements for groups ACLr, ACLa and CLL.

	**ACLr**	**ACLa**	**CLL**			
	**Passive**	**Active**	**Passive**	**Active**	**Passive**	**Active**
40°	44.7° ± 1.1°	40.8° ± 1.3°	44.2° ± 0.8°	40.6° ± 1.4°	44.7° ± 0.8°	39.9° ± 1.1°
90°	92.7° ± 1.3°	93.5° ± 1.4°	91.3° ± 0.7°	92.4° ± 1.4°	92.3° ± 1.0°	89.1° ± 1.0°

The median DIF at 90° position, for EG was 1.1°±0.9°, for ACLr and 1.2°±1.1° for ACLa. For CLL in the same position, DIF was 1.6°±1.0°. The median DIF in 40° position for EG, was 3.0°±0.9° and for ACLr 2.7°± 1.1° for ACLa. For CLL in the same position, the DIF was 3.2°±0.9°.

**Figure 4.**
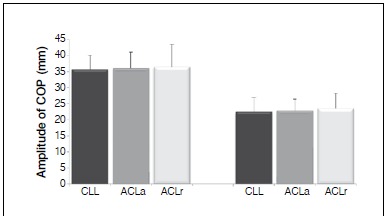
Mean values of amplitudes of center of pressure (COP) in the anteroposterior (COPap) and medial-lateral (COPml) positions, in unipodal support for groups CLL, ACLa and ACLr.

Figure 4 shows the results of the mean values of the maximum amplitude of COPap and the mean values ​of the maximum amplitude of COPml in unipodal position, for ACLr, ACLa and CLL groups. No significant differences were found for any of the two variables used to measure PC (COPap, p=0.950 and COPml, p=0.698).

**Figure 5.**
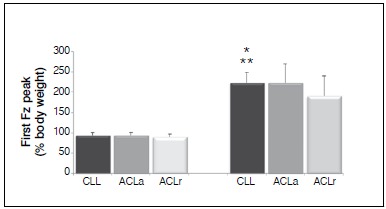
Mean values of first peak of the vertical component of ground reaction force (Fz) on climbing and descending the step for groups CLL, ACLa and ACLr. * Significative difference between ACLa e ACLr. ** Significative difference between CLL and ACLr.

In the SUD test, first peak values of Fz climbing the step in ACLr were on average 5% lower than ACLa and CLL values. However, this difference was not significant, for both comparison between ACLr and ACLa (p=0.180) and for the comparison between ACLr and CLL (p = 0.255). For descending the step the values ​​of the first peak of Fz of ACLr were on average 30% lower than ACLa and CLL values. The difference was significant for both ACLr and ACLa (p=0.035), and between ACLr and CLL (p=0.029). (Figure 5)

**Figure 6.**
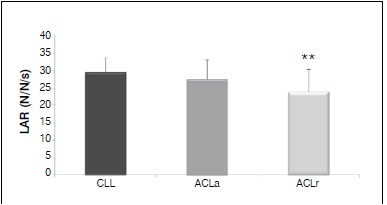
Mean values for load application rate (LAR) on descending the step, for groups ACLr, ACLa and CLL. ** Significant difference between CLL and ACLr.

The average of the LAR values (Figure 6) for descending the step was about 6% lower in ACLr group, indicating that the injured LL had less impact during the descent phase of the step than ACLa and CLL. These LAR values were significantly different between CLL and ACLr groups (p=0.008), but there was no significant difference between ACLr and ACLa groups (p=0.115).

## DISCUSSION

The question that guided the present study was whether individuals who underwent ACL reconstruction had sensorimotor changes six months after surgery. The main results demonstrated that individuals, after the postoperative period stipulated, had no proprioception and postural control deficits, but had motor deficits, influencing knee functionality.

The possible decrease in proprioception after an ACL reconstruction surgery is explained by the surgical removal of part of mechanoreceptors, especially Ruffini type endings and Pacinian corpuscles located in the skin and articulations.^7^^,^ 19 Improved proprioception at clinical evaluation is defined as a regeneration indicator of the joint receptors.^4^ Six months after surgery, there were no sensory deficits in proprioception, suggesting regeneration of ACL mechanoreceptors. Moreover, maintaining the present results, the PSE analysis report that morphologically normal mechanoreceptors can be found after three months of ligament reconstruction.^20^

From a clinical point of view, the literature is still controversial about the recovery of proprioceptive capacity after ACL injury.^3^^,^ 10 ^,^ 11 Studies evaluating knee proprioception found significant differences between injured and healthy knee at 45° bending angle six months after surgery.^13^^,^ 20 In contrast, other studies showed that proprioception returns to normal over the same period, justifying the recommendation for practicing normal activities of daily living.^1^^,^ 14

One possible cause for these contradictory results in proprioception response is the influence of the articular angle evaluated. Specifically, the recovery of proprioception was reported as satisfactory in extended (0-20°) and flexed (80-100°) knee positions, whereas at the intermediate angle (40-60°), proprioception levels were below normal values.^4^ Therefore, the present study confirms the greater difficulty in proprioceptive recovery for intermediary angles (40°), more than flexed position (90°) due to higher values of the DIF at ACLr at 40° angle compared to 90° (3.0° and 1.1°, respectively).

The extremes of the articular movement, as in the knee flexion and extension ranges, activate slow adaptation mechanoreceptors. These mechanoreceptors, the Ruffini endings, respond to passive movement and mediate the preparation of information on the member's position, allowing proprioceptive awareness.^4^^,^ 7 This proprioceptive awareness can also play an important role in knee protection, especially in extreme motion ranges.^4^

Proprioception is one of the most important sensory information to maintain postural control.^9^^,^ 21 Once proprioception is restored after ACL reconstructive surgery, the possibility of body oscillation is lower.^8^^-^ 10 ^,^ 22 The non significant differences in knee proprioception and unipodal postural control between LLs demonstrated a similar behavior between the two variables.

Although not significant, it was possible to notice a smaller amplitude of COPap and COPml for CLL compared to ACLr and ACLa. A possible explanation for this lower oscillation of CLL is that individuals from the EG, depending on the injury evidenced in ACLr, overload the healthy contralateral limb (ACLa), requesting it more often. This overload may lead to over-stimulation and consequent fatigue of ACLa, decreasing its performance compared to fully healthy knees from the CG.^10^ Compensatory overload can still increase the propensity of a recurrent injury in individuals undergoing their first ACL surgery or a new injury in the contralateral LL.^10^^,^ 23 ^,^ 24

The somatosensory system contributes with afferent information on body position to the CNS, generating, in turn, a motor response.^9^ Likewise postural control, functionality is part of the motor control and can also show positive correlations with proprioception.^3^ Mechanoreceptors present in the ACL are part of sensory system signaling an injury, interacting with other afferent signals to generate a sensation.^4^^,^ 25

This interaction with other sensory afferent, mainly muscle spindle, is able to explain the present findings, whose sensory deficits were not reported, but alterations in the motor control were found.^25^ Through the connection to the CNS, depending on the movement, inhibition of muscles motor neurons that surround the joint takes place.^7^

Joint receptors and muscle spindles assist with high efficiency the JPS, however, the interpretation of afferent signal requires information on the motor command sent by the muscle. The motor command or effort signals contribute to the position awareness, force and segment motion. Depending on the motion requirement, compensatory muscle strategies may be being adopted to restrict, compensate and protect the injured knee.^25^ When normal afferent signals are evaluated by the CNS, the restriction of some movement is possibly related to the muscle motor control due to protection of the injured segment.^25^

The SUD test examines a functional activity, by assessing the sensorimotor control and force.^16^^,^ 26 During this test, the assessment of force values upon climbing the step quantifies the concentric force to execute such a movement and lead the contralateral leg. A good force index to climb the step is indicative of good ability to force production during the concentric contraction of the quadriceps.^16^

Previous research reported that muscular force deficits can be found from six to twelve months after ACL reconstruction surgery, mainly in the quadriceps and hamstrings. 11 Changes in muscle force may be due to activation failure or muscle atrophy, reaching deficits up to 6-10% in relation to the contralateral leg.^17^ In the SUD test, the values of the first Fz peak for ACLr climbing the step were 5% below those of contralateral LL control values. This result may have been found due to decreased muscle force in ACLr, confirming previous studies that found a significant difference between LLs in vertical force behavior evaluated in force platforms while performing functional activities.^16^^,^ 27

During the descent of the step in the SUD test, the impact is quantified the impact of the LL at the movement landing. A high impact descending the step is indicative of lower motor control of the LL driving the movement. Likewise, it is possible to check the eccentric ability of the LL that is leading the other down the step. In the case of a weak LL to lead the movement, the impact on the descent is high. In contrast, in the case of a strong LL, it is able to control the motor action more efficiently, reducing the impact on the contact surface with the ground.^16^

Moreover, previous researchers reported that LLs joints contribute to the absorption of impact forces during the landing movement by energy dissipation. This ability to dissipate impact forces is different when the landing is performed unipodal and bipodal. In the frontal plane in bipodal support, the hip is primarily responsible for the total energy dissipated, corresponding to 66.7%, while the knee dissipates 29%. In contrast, in unipodal landing, energy dissipation by the knee increases to 60.7% and the hip contribution lowers to 36.6%.^28^

In the case of the hip joint, muscles are responsible for maintaining stability, however, in the knee, the impact is absorbed mostly by passive structures, such as ligaments, maintaining the joint stability.^28^ The ACL is a major ligament of the knee and, hence, one of the main responsible for absorbing impact in such situations.^1^ Good knee movement during landing is associated to increased joint energy dissipation. However, when there is any anatomical limitation of the joint, other movement strategies should be adopted in order to improve shock absorption and reduce the risk of injury.

Significant reductions in knee flexion movement were observed six months after surgery that may also be viewed in step climbing test.^28^ This flexion decrease on the LL leading the descending movement suggests a greater application of force and impact in the LL that touches the ground.

These statements support the significant decrease in first Fz peak value and LAR for descending the step, in a landing situation. Thus, six months after ACL reconstruction, force changes to perform the movements remain, knee flexion amplitude and signaling peripheral damage is maintained, protecting the joint. With this reduction in motor control, the measured force values are altered.

Many studies also reported functional deficits after ACL surgery,^3^^,^ 8 ^,^ 11 but they used other assessment protocols. Several functional tests are used to measure the performance of the lower end, such as unipodal vertical jump or distance jump.^23^ These tests are recommended for post-surgical athlete evaluation, since they are considered similar to demands of top-level sport and, therefore, more challenging than walking or running. However, this test has a high impact level, and it is not advised to non-athlete individuals with low post-operative time, and therefore, the proposal for other functional tests, such as SUD and AAF.^15^

Functionality testing has also allowed to infer persistence of movement deficits and muscle force in the knee joint. However, further studies with other methodological techniques are indicated, such as isokinetic dynamometers^8^ to assess muscle force, or kinematics^23^^,^ 28 to assess range of motion.

Finally, it is worth noting that even rehabilitation clinics recommending a patient follow-up of six months after ACL reconstruction, a thorough investigation is recommended to evaluate possible sensorimotor changes thereafter. This is because, with the present findings, motor abnormalities are still found, requiring motor control recovery for a longer period.

## CONCLUSION

The results presented in this study showed that individuals who underwent ACL reconstruction surgery after a six-month postoperative period showed no change in proprioception and postural control, but presented changes in motor control, influencing knee functionality. More studies need to be performed to clarify other possible motor changes and investigate the relationship of the findings to different surgical and rehabilitation techniques.
